# The Impact of Caregiver Burden and Behavioral and Psychological Symptoms of Dementia on Patient Healthcare Costs in the United States

**DOI:** 10.1002/alz70858_099329

**Published:** 2025-12-25

**Authors:** Lauren Chrzanowski, Landon B Peeples, Benjamin Mast

**Affiliations:** ^1^ University of Louisville, Louisville, KY, USA; ^2^ University of Louisville, Louisville, KY, USA

## Abstract

**Background:**

Recent literature suggests caregiver burden may influence healthcare costs more than symptomatology. The current study investigated the relationships between behavioral and psychological symptoms of dementia (BPSD), caregiver burden, and three patient healthcare cost outcomes: (1) medication, (2) hospitalization, and (3) outpatient visits.

**Method:**

Data were analyzed from the Longitudinal Cohort Study of Resource Use and Cost of Mild Cognitive Impairment and Mild Dementia Due to Alzheimer's Disease in the United States (GERAS‐US), a retrospective cohort study. Participants (*N* = 1,476) with early Alzheimer's dementia and a collateral study partner (e.g., family caregiver) were contacted across six timepoints from 2017‐2021. Linear mixed‐effects models were fit to data using Restricted Maximum Likelihood (REML).

**Result:**

The first model evaluated patient medication costs. Significant effects were observed for caregiver burden (Zarit Burden Interview scores; *b* = 5.67, *p* < .001) and frequency of BPSD (Neuropsychiatric Inventory scores): depression/dysphoria (*b* = 48.64, *p* < .001), anxiety (*b* = ‐68.35, *p* < .001), irritability/lability (*b* = ‐81.36, *p* < .001), and apathy/indifference (*b* = ‐49.28, *p* < .001). Variability across visits was significant (*σ* ^2^ = 12229), with high residual variance (*σ* ^2^ = 150564).

The second model assessed hospitalization costs. Caregiver burden (*b* = ‐4.80, *p* = 0.016) and BPSD, including depression/dysphoria (*b* = ‐103.18, *p* = .010) and irritability/lability (*b* = 90.57, *p* = .011), were significant predictors. Anxiety and apathy/indifference were not significant predictors (*p's* > .05). Variability across visits (*σ* ^2^ = 54303) was smaller than residual variance (*σ* ^2^ = 1327584).

The third model examined outpatient visit costs. Anxiety (*b* = ‐28.79, *p* < .001), apathy/indifference (*b* = 14.87, *p* < .001), and irritability/lability (*b* = 22.12, *p* < .001) were significant predictors. Depression/dysphoria and caregiver burden were not significant predictors (*p's* > .05). Variability across visits was moderate (*σ* ^2^ = 1973) compared to residual variance (*σ* ^2^ = 14440).

**Conclusion:**

Findings indicate that caregiver burden and BPSD significantly influence patient healthcare costs, though their impact varies by cost category. Targeted interventions to mitigate caregiver burden and manage BPSD could reduce healthcare costs and improve outcomes. Addressing these factors remains an important clinical and research priority.

